# Supplementary material for: Performance of the Abbott Architect Immuno-Chemiluminometric NT-proBNP Assay
Source: Diagnostics (Basel). 2022 May 8;12(5):1172. doi: 10.3390/diagnostics12051172 (PMC9140882; doi:10.3390/diagnostics12051172)
Supplement: Supplementary file 1 [file diagnostics-12-01172-s001.zip › diagnostics-1658171-supplementary.pdf]

Supplementary Table S1: STARD checklist

Section & Topic	No	Item	Reported on page #
**TITLE OR ABSTRACT**			
	**1**	Identification as a study of diagnostic accuracy using at least one measure of accuracy (such as sensitivity, specificity, predictive values, or AUC)	**2**
**ABSTRACT**			
	**2**	Structured summary of study design, methods, results, and conclusions (for specific guidance, see STARD for Abstracts)	**2**
**INTRODUCTION**			
	**3**	Scientific and clinical background, including the intended use and clinical role of the index test	**4**
	**4**	Study objectives and hypotheses	**6**
**METHODS**			
Study design	**5**	Whether data collection was planned before the index test and reference standard were performed (prospective study) or after (retrospective study)	**7**
Participants	**6**	Eligibility criteria	**7**
	**7**	On what basis potentially eligible participants were identified (such as symptoms, results from previous tests, inclusion in registry)	**7**
	**8**	Where and when potentially eligible participants were identified (setting, location and dates)	**7**
	**9**	Whether participants formed a consecutive, random or convenience series	**7**
Test methods	**10a**	Index test, in sufficient detail to allow replication	**7**
	**10b**	Reference standard, in sufficient detail to allow replication	**7**
	**11**	Rationale for choosing the reference standard (if alternatives exist)	**7**
	**12a**	Definition of and rationale for test positivity cut-offs or result categories of the index test, distinguishing pre-specified from exploratory	**7**
	**12b**	Definition of and rationale for test positivity cut-offs or result categories of the reference standard, distinguishing pre-specified from exploratory	**7**
	**13a**	Whether clinical information and reference standard results were available to the performers/readers of the index test	**7**
	**13b**	Whether clinical information and index test results were available to the assessors of the reference standard	**7**
Analysis	**14**	Methods for estimating or comparing measures of diagnostic accuracy	**9**
	**15**	How indeterminate index test or reference standard results were handled	**9**
	**16**	How missing data on the index test and reference standard were handled	**9**
	**17**	Any analyses of variability in diagnostic accuracy, distinguishing pre-specified from exploratory	**9**
	**18**	Intended sample size and how it was determined	**Not applicable**
**RESULTS**			
Participants	**19**	Flow of participants, using a diagram	**Not applicable**
	**20**	Baseline demographic and clinical characteristics of participants	**7**
	**21a**	Distribution of severity of disease in those with the target condition	**Not applicable**
	**21b**	Distribution of alternative diagnoses in those without the target condition	**Not applicable**
	**22**	Time interval and any clinical interventions between index test and reference standard	**Not applicable**
Test results	**23**	Cross tabulation of the index test results (or their distribution) by the results of the reference standard	**10**
	**24**	Estimates of diagnostic accuracy and their precision (such as 95% confidence intervals)	**Not applicable**

	**25**	Any adverse events from performing the index test or the reference standard	**Not applicable**
**DISCUSSION**			
	**26**	Study limitations, including sources of potential bias, statistical uncertainty, and generalisability	**18**
	**27**	Implications for practice, including the intended use and clinical role of the index test	**17**
**OTHER INFORMATION**			
	**28**	Registration number and name of registry	**9**
	**29**	Where the full study protocol can be accessed	**Not applicable**
	**30**	Sources of funding and other support; role of funders	**Not applicable**

Supplementary Table S2: Within-run precision of the Abbott NT-proBNP assay at both sites.

Level	Range	Mean	SD	CV%
Site A (Singapore)				
L1	130.1-147.1	137.6	3.91	2.84
L2	475.0-541.4	505.7	16.0	3.16
L3	4678-5434	4980	179.8	3.61
Site B (Netherlands)				
L1	-	142.0	6.10	4.30
L2	-	506.3	11.0	2.17
L3	-	4973	124.5	2.50

Supplementary Table S3: Between-run precision of the Abbott NT-proBNP assay at both sites

Level	Range	Mean	SD	CV%
Site A (Singapore)				
L1	129.3-154.1	139.5	5.82	4.17
L2	486.0-560.5	521.6	20.0	3.83
L3	4646-5473	5053	232.6	4.60
Site B (Netherlands)				
L1	-	142.0	6.39	4.50
L2	-	506.3	11.0	2.17
L3	-	4973	124.7	2.51

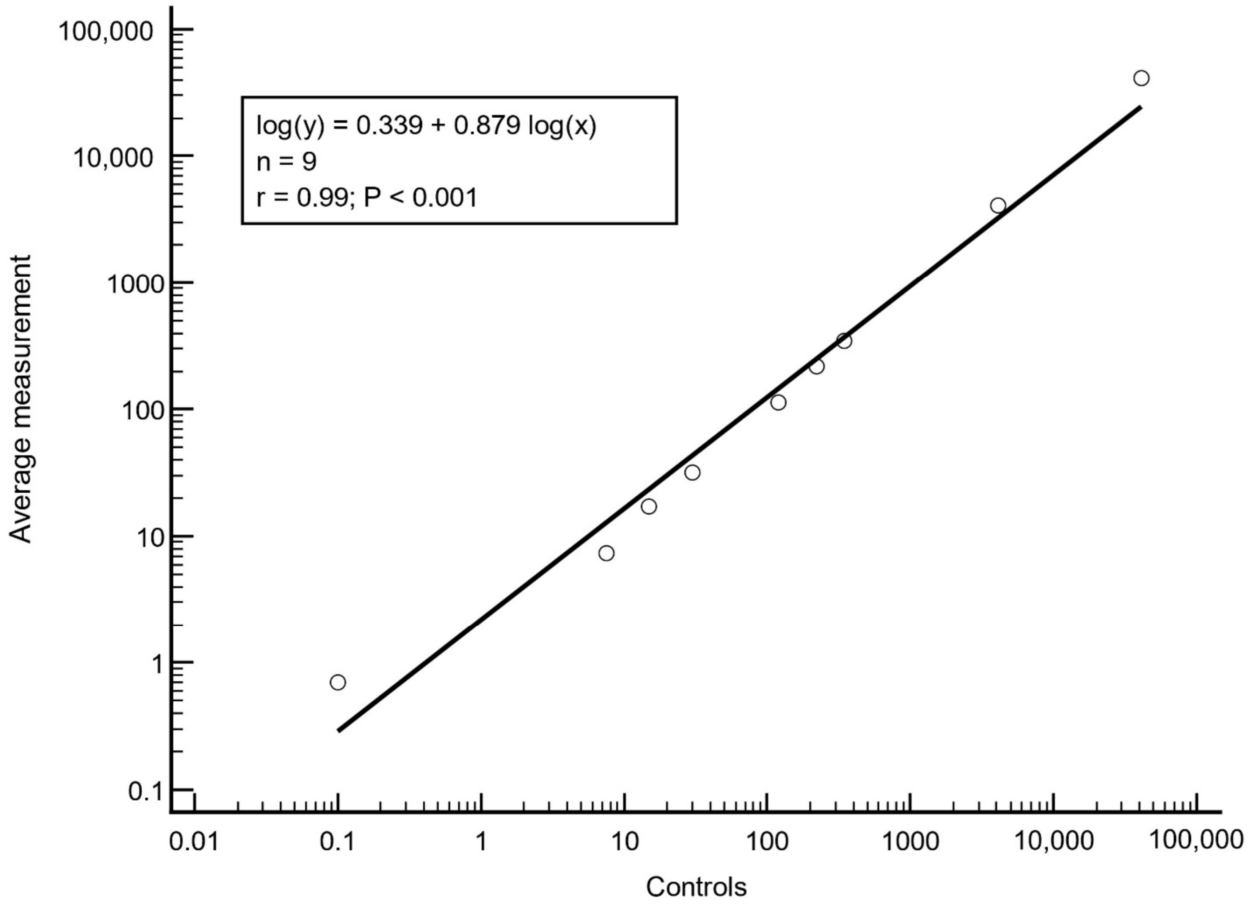

Supplementary Figure S1: Linearity analysis of the Abbott NT-proBNP assay at Singapore site.

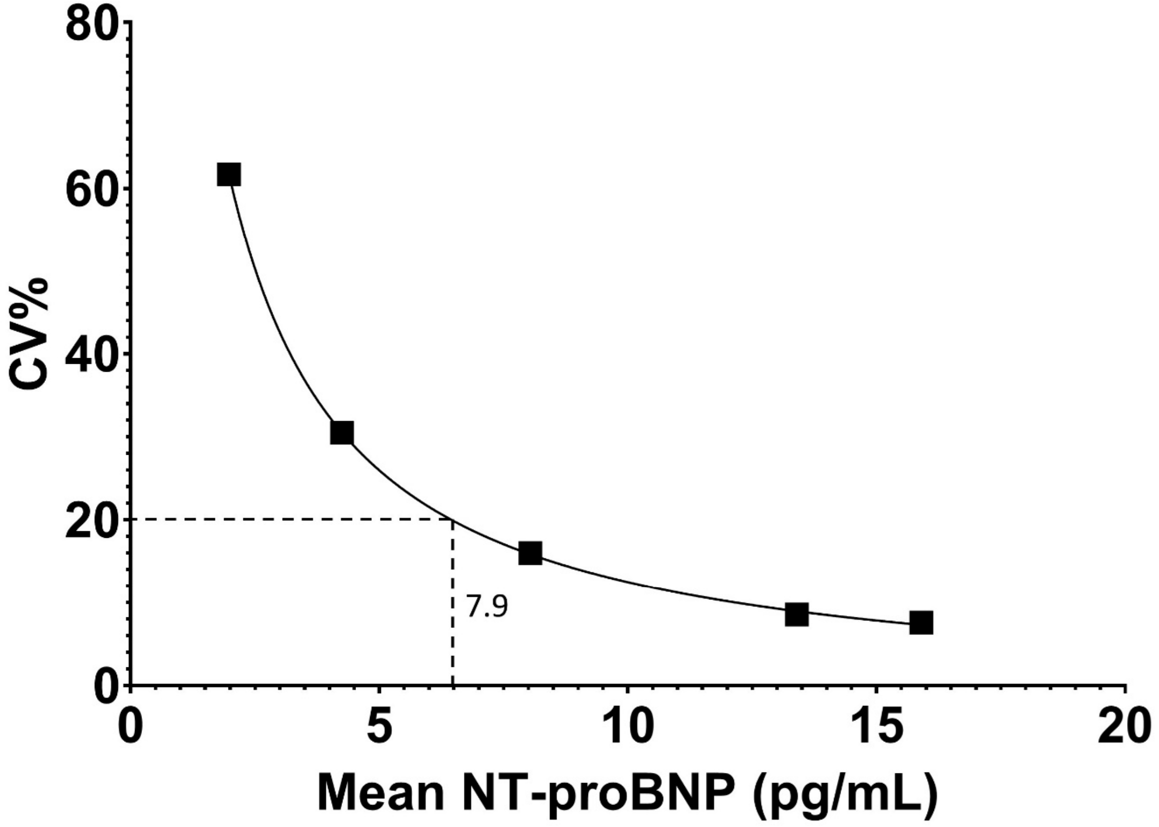

Supplementary Figure S2: Limit of Quantitation analysis of the Abbott NT-proBNP assay at Singapore site. 20% CV corresponded to a value of 7.9pg/mL. Abbreviations: NT-proBNP: N-terminal pro-brain natriuretic peptide
